# Attention-Setting and Human Mental Function

**DOI:** 10.3390/jimaging8060159

**Published:** 2022-06-01

**Authors:** Thomas Sanocki, Jong Han Lee

**Affiliations:** Department of Psychology, University of South Florida, Tampa, FL 33620, USA; lee84@usf.edu

**Keywords:** attention, visual attention, human perception, human cognition

## Abstract

This article provides an introduction to experimental research on top-down human attention in complex scenes, written for cognitive scientists in general. We emphasize the major effects of goals and intention on mental function, measured with behavioral experiments. We describe top-down attention as an open category of mental actions that initiates particular task sets, which are assembled from a wide range of mental processes. We call this *attention-setting*. Experiments on visual search, task switching, and temporal attention are described and extended to the important human time scale of seconds.

## 1. Introduction

Human visual cognition examines specific mental functions, using behavioral measures. Our aim here is to leverage 6+ decades of experimental research in visual cognition and attention, to present a picture of the way the brain is cognitively “active” during everyday perception: the top-down implementation of intentions and goals, realized by attention-setting. Triggered by goals such as *find (object)*, attention sets up and prioritizes relevant machinery in neural subsystems. These are not the only top-down effects in the brain, but they appear to be the most powerful and important.

We present *attention-setting* within a general treatment of attention that emphasizes visual tasks in complex scenes. We begin with some historical background and then develop the concept of attention-setting in tasks such as visual search. This is followed by a selective review of major experimental results on task-switching and temporal attention. Then, we develop the idea of attention-setting over seconds. This time scale is especially important in human behavior [1]. We present evidence that attention is especially powerful when it can be set and changed over seconds. We finish with some relevant highlights from neuroscience and cognitive health. Our general argument is that attention-setting is a pervasive top-down mental skill, which plays out over multiple timescales, especially the humanly important time scale of seconds.

### 1.1. Pieces of History

A basic principle of attention is that perceivers choose one stream of information out of many; this is a main way in which the brain is active. This idea was first explicated by William James in 1890 [2] and has been re-stated in recent decades [3,4]. The brain is limited, in contrast to the immense informational richness of any real-world situation. Furthermore, computational studies demonstrate that interpretation is exponentially more complex than the stimulus, an even stronger motivation for attention [5,6].

In the early days of scientific psychology in the 1890’s, attention was a main topic (e.g., [7,8]). Effective empirical approaches such as “task switching” were being developed into the 1920’s [9]. However, when behaviorism became dominant in the 1930s and 1940s, attention studies were not published in primary experimental research journals. Attention was not directly measurable. The cognitive approach to attention re-appeared in the 1950s as internal mental constructs, and the evidence-based inferences necessary to support them were again allowed in the research mainstream. Since that time, the field of attention has developed and flourished. Major results have emerged and been replicated many times, producing some areas of general empirical agreement.

The larger world-context in the 1950s was the dawning of the information age and ideas such as measuring information and manipulating it. Attention researchers could present different types of information and infer internal mechanisms from the resulting patterns of human performance. In the first major theory, attention was hypothesized to be an *early filter* that selected among sensory information coded in parallel across sensory registers. Consequently, information matching a single sensory register (a “channel”) should be easy to select and then process further into later, deeper perception [10,11]. With the then-modern technologies of tape recorders and headphones, researchers used broad sensory channels such as input ear, and difficult tasks such as dichotic listening: repeating one “relevant” message (e.g., words in the left ear) while ignoring other, irrelevant messages (words in the right ear). The early filter construct was supported by the result that repeating an input stream was efficient when it was distinct on a sensory basis (e.g., high voice in one ear) but less efficient when sensory selection was difficult (e.g., relevant inputs switched ears, or high voices in both ears) [10,11].

The second major theory moved the filter later into the processing stream, positing a *late filter* after basic perceptual identification had been completed. Objects were identified in parallel, and the filter was used to select a single identified object for admission to limited consciousness (e.g., [12,13]. As Kahneman and Treisman noted in 1984 [14], early and late selection were scientific paradigms in the Kuhnian sense [15]; the paradigms provided theory together with methods. The late selection methods tended to favor efficient processing; the stimuli were familiar and simple (e.g., letters in arrays), as were the tasks (e.g., *detect a T or F*), which encouraged late processing. The early selection methods, on the other hand, involved broad channels (such as ear) and difficult tasks that encouraged early section [14]. Ultimately, the early versus late debate was resolved with hybrid models in which each type of selection could occur, but with important differences such as the relative ease of early selection and the broadness of late selection [16,17].

Note that attention was used mainly as a noun, denoting a mechanism. The early decades of research can be viewed as a search for *the* mechanism of attention, as well as a search for manipulations that could separate attention from other processes, such as perception. Kahneman and Treisman [14] acknowledged the fruitfulness of the filter metaphors but proposed a framework that was literally more integrative. Perception and attention were functionally related in the key construct of mental “object-representations”. A mental object can be thought of as a top-node in a perceptual hierarchy. Object representations efficiently integrated perceptual, conceptual, and event information, and this produced a priority for unified mental concepts. The object-metaphor was also quite fruitful and generated considerable research. The functional relationship between perception and attention was new to this attention framework, although some perceptual theorists of the 1970’s suggested a similar relationship [1,18,19]. The functional relationship continues in most modern conceptions, including the present one.

As attention research grew over the next decades, the range of attentional functions studied in experiments greatly increased. Franconeri [20] describes and explains 15 different attentional limitations within a common framework. Geng, Leber, and Shomstein [21] recently called for research articles on attention and perception and published what they termed 40 different views. Research has also begun to address the complexity of real-world situations, which magnifies the importance of attention and priority (e.g., [6,22,23,24]). Situations that approach real-world complexity are emphasized here.

Over the decades, the theoretical metaphors of attention became less singular and more general, each capturing important aspects of attention: a single pool of energetic resources (e.g., [25]), multiple pools with distinct resources (e.g., [26,27]), the object-centered structures mentioned [14], attentional sets (e.g., [28,29]), and biased competition between representational networks [30,31,32,33]. The multiplicity of concepts suggests that the functions of attention are too varied and too pervasive to be captured by any single mechanism.

### 1.2. Attention-Setting

Attention-setting is a set of skills, i.e., mental actions. Attention-setting is a verb phrase designating the category of mental actions that initiate and prioritize mental functioning within the limited resources of the human brain. Attention sets up mental processing in accordance with the observer’s goals and situational parameters. Once set up, familiar processes such as “read that highway sign” run as a continuing interaction involving the mind, the display, and the larger situation. We illustrate attention-setting further with an example involving the well-studied process of visual search. Attention-setting extends over seconds in the example, consistent with our emphasis on humanly important time scales [1]. Theories and evidence supporting this example are noted below. In the section that follows, we relate attention-setting to the theoretical concepts in the literature and illustrate the pervasiveness of attention.

Theory and Evidence behind the Example
A comprehensive theory of visual search has been developed by Wolfe and colleagues and provides details on many important visual mechanisms—Guided Search Version 6.0 [34]. The theory integrates well-supported details of sensory coding channels and the priority map, and the paper provides useful further references. Zelinsky, Chen, Ahn, and Adeli [35] provide an amazing catalog of computational search models, with an emphasis on the general problem and real-world scenes. Zelinsky et al. treat eye fixations, which are closely linked to attention; they provide a complementary theoretical approach to top-down influences (see also [36]).
Attentional templates are included in most search models, and many experiments measure the set-up of the templates. Priority maps are also central constructs; they combine top-down knowledge and visual features from bottom-up parallel processing (e.g., [34,37,38]). Priority maps are used to guide the search toward likely target locations and away from unlikely locations [39]. The trade-offs in energetic resources between different tasks is a long-standing topic in basic and applied research (e.g., [27,40]). Internal attention, such as turning attention into one’s memory, is becoming a distinct research topic (e.g., [41,42]). Unconscious problem solving is a growing area of research. The idea that processes will be modified over time, through interaction with the world, was proposed by Neisser [1] as the perceptual cycle. We will develop the idea below as a useful framework for understanding attention over the time scale of seconds.

Imagine that the power went out at night and it is near dark in one’s home. Intention takes the form of a goal such as *find (flashlight),* and attention sets up processes to meet the goal. Attention sets up visual search processes by initiating the creation of an internal attentional template for the goal object (target), in visual working memory. The template can be fairly specific (*my red flashlight in dim light*) or abstract in various ways (*any light source*). The template is used in a matching process that compares it to a priority map of the visible world. The priority map combines sensory information (bottom-up features) and knowledge (top-down) on a spatial map. The sensory features in the map are weighted by priority (e.g., *reddish glints of light, non-accidental shape properties*). The knowledge includes historically likely locations (*the flashlight should be on its shelf*). The attentional template is matched against the priority map, to guide the search through the immediate scene. In near darkness, the search may be slow because the incoming features are limited by low light (a data limitation; [25]). Because bottom-up information is weak, top-down location knowledge will be more important, but only if valid (*and only if the flashlight has been put back on its shelf*).

Once the search process has begun, it will continue to require some mental resources but usually less than at the start. In addition, attention can set up new processes such as reaching or tactile search, again drawing on resources. Attention can also set up internal processes. It can initiate wider problem solving, including memory retrieval, which is set up with a memory cue (e.g., *when did I last use the flashlight?*). The results of these processes (*when I was fixing the toilet*) can then be used to modify priorities. Problem solving is aided by abstract goals (*find the light*) and is set up by attention; a goal can serve as a memory cue that can activate unconscious knowledge. *Phones now have flashlights.* The activation of an unconscious memory is not directly caused by intention; activation is caused because an abstract goal (memory cue) is broadcast to memory and there is a match.

In sum, attention sets up and guides processes at multiple levels and modifies priorities as results come in from the world, decision making, and memory. Attention sets up, guides, and prioritizes larger systems (e.g., visual search, tactile search, and memory retrieval), initiates the construction of central objects within systems (e.g., attentional templates and retrieval cues), and implements priorities at multiple levels (e.g., favoring particular tasks, locations for search, and certain visual features, while inhibiting unlikely features and locations). The exact number of attention-setting functions may be difficult to know because humans invent and tune new cognitive skills. Nevertheless, we think that attention-setting could explain the major goal-directed mental actions of the perceiver, across many situations.

The boundary between attention and other mental processes is an interesting issue. We argue that a strict boundary is not yet appropriate for attention. A more fruitful approach is to assume that attention-setting works directly with other processes and examine those functional relationships. At today’s levels of discovery, functional relationships are more important than carving mental nature into independent parts.

Attention-setting is an expansion of attentional set theory, which has emphasized specific sets within controlled situations (e.g., [23,28,43]). We expand upon this idea, arguing that that attention-setting is a powerful set of skills involving setting and tuning. Setting often takes place over seconds, during interactions with the world. As we explain later in this paper, the settings of attention can have profound effects. Because set helps determine the information that observers pick up from the world, set also helps determine what observers understand and learn [1].

### 1.3. Relations to Other Major Concepts and Processes

The mental resources prioritized in attention-setting are often called “attention” in the literature, for simplicity. Resource limitations are critical (e.g., [25,26,27]), and attention-setting is constrained by the limitations. “Attending” is a basic result of attention-setting. Attention-setting is similar to the widely studied construct of attentional control (e.g., [6,44]). Attentional control is a fundamental executive process in the brain (e.g., [45]). However, attention-setting emphasizes the setup of brain processes to run rather than continuous control. Set-up (preparation) is often a highly resource-intensive process (e.g., [46]).

Attention-setting (and attention in general) is functionally related to many mental processes, and we will now mention some of the most important. Extending upward, there is the executive domain of meta-awareness and executive processing, where initiating and setting processes is often critical (e.g., see [47]). Attention in general is closely related to awareness; Graziano’s attention-schema theory provides good treatment (e.g., [48]). Attentional control is a major portion of intelligence, and attention-setting may be a core mechanism in the portion termed *fluid intelligence*, the flexible, creative, and problem-solving aspects of intelligence [44,49]. Skillful attentional control is necessary for creativity and imagination. Attention sets up mental “simulations” that involve knowledge and images assembled from memory and that may seem to run themselves as long as they continue to be attended (e.g., [50,51,52]). 

Attention-setting works with each of the main types of memory. It is functionally related to working memory, which is a highly flexible, temporary representational space. Attention sets up and uses working memory in multiple ways, for example, as an image-like memory buffer, or a verbal rehearsal mechanism to remember a set of numbers [53]. Attention-setting also interacts with long-term memory, by setting up cues broadcast to memory (e.g., [54]). In fact, memory retrieval can be viewed as attention-setting turned inward [42]. Third, attention-setting is initially critical for developing implicit memory skills such as driving. Beginners set up the new tasks carefully in a serial, attention-controlled (and resource-intensive) manner. However, with practice the procedures become an implicit memory that runs with low resource requirements. The links between intentions and the networks that implement them are critical, and recent work has begun to flush them out conceptually and formally (e.g., [55,56]). In summary, attention-setting contributes to many mental processes, and these functional relations are active research topics.

### 1.4. The Present Approach

The strongest arguments for the attention-setting framework come more from the “big picture” of attention than from any single experiment. We believe attention-setting is consistent with many of the thousands of experiments on top-down attention. Furthermore, critical support also comes from success in related fields, when attention is viewed as an active and pervasive top-down influence on networks. This includes research on attentional disabilities [57] and computational vision [6]. Near the end of this paper, we will bring in evidence from neuroscience and the emerging sciences of attentional and cognitive health and note that attention-setting has biological characteristics such as exercise-benefits and fatigue.

The overall goal in this paper is to provide an informative but highly selective tour of attention and attention-setting in the field of visual cognition. We use verbal and descriptive concepts typical of the field and emphasize relatively complex situations that begin to resemble the real world. The aim is to capture the most important messages from recent decades of experimental research, in a “consumer-friendly” manner. That should mean readable, but in psychology and neuroscience, the units readers care about most are “effect sizes”. How large is the effect of attention-setting on mental performance? Visual cognition provides some scales that are simple and intuitive. Here, our favored scale is the ability to perceive something in plain sight, such as a gorilla. Usually performance is near 100% for this process, but research puts some interesting marks on the other side of the scale.

## 2. Attention-Setting and Gorilla Missing

The missed-gorilla experiment is a landmark demonstration in the domain of attention [58]. We will describe that experiment, but first readers should note that they can still experience misperception in the original video, or experience it anew in the sequel, “Monkey Business” (http://www.theinvisiblegorilla.com/videos.html (accessed on 10 June 2021)).

The original experiment demonstrates the powerful effects of attention-setting over seconds. As mentioned, noticing a gorilla is usually near 100%, even in video. However, this ability is greatly reduced when healthy observers engage in a visually and mentally challenging task while watching a video with two interacting teams of players. In a representative condition, there were two teams of three players each (white shirts versus black shirts), and the task was to notice passes of a basketball by one team (task focus 1) and count the number of passes (task focus 2). The players in the other shirts should be ignored (suppression; task 3). This makes the observers busy, maybe as busy as crossing a city street. The critical finding was that when the gorilla walked in and pounded his chest, only 42% of observers reported noticing it when subsequently asked, “Did you notice anything else?”. The results have stood up to years of scrutiny, including careful considerations of memory [43], and further research, including more controlled conditions to be described. Because false alarms were low (no false gorilla reports by another group of observers), the hit rate is a valid scale of conscious perception. The missed gorilla is a marked failure of the mental processes that lead up to conscious perception, a failure that lasts for seconds. There is likely to be limited unconscious processing in this situation, however, as will be noted.

A reasonable explanation for the conscious failures is that the attention settings were for the relevant task, pass-counting. The settings enable processes for the three challenging foci mentioned above, beginning with the complex processes of tracking complex objects in space (both the ball and white-shirt players). This requires guidance systems for eye movements and attentional resources, as well as the executive direction of counting and remembering. The *task set* also includes the suppression of non-relevant information and especially the black-shirt players. Interestingly, when the colors are reversed for other observers (attend to black shirts, ignore white), the color-settings change and the gorilla is noticed 83% of the time. However, for other subjects, the gorilla is replaced by a woman wearing light grey clothes with an umbrella, and she is noticed only 58% of the time.

Thus, the results are not due to a single mechanism but instead a configuration of systems, the task set. The task settings are also likely to pertain to time and size scale; the basketball is the primary object and is relatively small and fairly fast, in contrast to the slower and larger unexpected people in guises. The configuration of systems gives the set selective high efficacy in the relevant task but causes the human to miss many other stimuli outside of the set. Noticing an unexpected stimulus requires bottom-up capture, to be described.

### Gorilla Missing with More Control, and Bottom-Up Capture

Attention-setting is an internal action that changes the functioning of the brain. The match or mismatch between the settings and the experimenter’s stimuli can produce large differences in performance. Researchers can observe the match and mismatch by changing the task, and do so repeatedly (100s of times) to obtain more reliable data. When the task changes back and forth repeatedly, participants learn to change settings with some efficiency; this is known as *task switching* or *task reconfiguration*. This has been an intense area of research (e.g., for reviews see [59,60,61]), and we will be switching back to it throughout this paper. Note that in task switching research, the search for *the* mechanism (a single structural bottleneck) may be successful only in limited situations (cf. [62]). Perspectives of flexibility and practice are necessary to explain major results [59]. Thus, we return to the first switch in task, which usually produces the largest change in mental function.

Observing the first matches and mismatches of set requires a special type of experiment, usually one without task-specific practice that stabilizes performance. Findings such as missed gorillas helped inspire an era of these experiments that led to important insights. Gorilla missing with more controlled displays was measured in a program of experiments led by Steve Most, who was a graduate student drafted onto the Simons and Chabris gorilla team. Together, they directed an army of researchers with laptops far and wide across campuses, to conduct dozens of short experiments [29,63]. 

In a number of experiments, the stimuli were 8 smallish black or white circles and squares, about the size of a large-ish coin [29,63]. The shapes moved haphazardly across the display screen over seconds. Depending on the experiment, the task set was to track 4 of them, defined by color or by shape, and to ignore the 4 others. When squares were tracked (black *and* white), attention set a visual-cognitive “square-template” for the relevant squares while inhibiting irrelevant circles. The role of the gorilla was played by an unexpected ninth object that entered the screen. When attention was set for squares, observers noticed a square intruder much more often than a circle-intruder. In another experiment, the template for relevance was “black” (or “white”), and observers tracked that color. The gorilla was played by a cross similar in size, and either black, white, or one of 2 intermediate levels of grey. The cross was almost always noticed when it was the attended black or white color (93%), but noticing went down linearly the next 3 grayscale steps away, to 3% with maximum departure (e.g., black relevant; white cross). This function, spanning most of the range in performance scale, wins a prize for the largest effect size in this paper.

Another important special paradigm was devised by two sages of the cognitive revolution, Ariel Mack and Irvin Rock [64]. They sought to measure perception that was unprepared and low on directed attentional resources. The observers’ efforts were directed to a briefly appearing cross, for which they would compare the length of the two segments (to establish if the horizontal or vertical was longer). For 3 trials, only the cross appeared but briefly, so the observers were set for optimal size processing. On the fourth trial, the cross appeared again but along with a nearby, unexpected stimulus. Would observers notice a simple but unexpected stimulus such as a line or colored shape? Across many experiments, a variety of simple stimuli went unnoticed by most observers, even though the stimuli should activate simple feature detectors in the observers’ brains. At this point, the results were quite pleasing to a hard-core top-down theorist: even simple stimuli were not perceived, if the observer was not set for them.

However, researchers keep on experimenting, and the simple conclusion was qualified with an important twist. Mack and Rock [64] found that if the unexpected stimulus was more meaningful—the observer’s printed name—most observers (87%) noticed it. The finding echoes a now-classic finding that seriously hampered the early filter model, concerning information from an unattended (ignored) ear during dichotic listening. If selection was sensory-based, then everything in the ignored sensory channel was thought to simply decay; indeed, participants remembered nothing from that ear. Then, Moray [65] found that the participant’s own name could be noticed and remembered. Thus, unexpected but significant signals may be processed into awareness, through a primarily bottom-up route. Classic theories of attention added mechanisms for prioritizing significant information (e.g., [66]). More recent research indicates that this critical result has narrow boundary conditions, however [67]. More generally, as theorists recognized in the 1970s, the flow of information during perception is both bottom-up and top-down in nature (e.g., [19,25]). Humans like to be driven by their knowledge, but adaptation requires being open to unexpected inputs and new ideas. Modern theories include rapid bottom-up routes for efficiently processing familiar stimuli, along with more controlled top-down mechanisms (e.g., [68,69,70]). In fact, a possibly major difference between individuals is the degree to which a person is top-down or bottom-up in general [71].

However, in a more precise sense, the relative strength of purely bottom-up routes and top-down settings remains a critical issue. The ability of an unexpected but physically salient stimulus to capture attention is a demonstration of the power of bottom-up processing (e.g., [72,73]). For example, observers set to respond to blue-Ts can be slowed by a nearby but “irrelevant” red-X. This is at least somewhat independent of top-down settings. However, note that observers in such studies form general sets, such as using vision and responding rapidly to sudden stimuli. In the now large literature on this topic, critical factors include the spatial region to which attention is set and the degree of task-relevance of the stimulus (see [74], this issue). Bottom-up capture can be eliminated in some general conditions, for example, with an exclusionary attentional set such as “ignore red” or “ignore that region” (e.g., [75,76,77]). Thus, in many cases, the capture of attention is contingent on high-level settings (e.g., [78]). This is a critical indication of the power of top-down processing. Purely bottom-up capture appears to be limited to certain experimental conditions [79].

Nevertheless, bottom-up processing routes are efficient for a variety of stimuli, from familiar words to novel but typical everyday scenes. Additionally, some information is prioritized, including negative information and self-relevant information (e.g., [80,81]). Some processing is unconscious. For example, if a human is set to watch a certain region of the video screen and an unexpected but familiar word appears near there, it is likely be processed to some depth in the brain, independent of other ongoing processes. The familiar word activates feature, letter, and word detectors in intermediate brain areas, resulting in some activation of meaning (cf. [30,82,83]). This can happen while an observer’s awareness is focused on another task. Such effects qualify the large effect-sizes of tasks set on mental function that we have emphasized. When a stimulus such as a gorilla or a circle in clear view is missed, there is likely to be some stimulus-specific processing at unconscious levels.

The research discussed so far has focused on limited windows of time—single critical trails and events, either in the first parts of experiments or in some cases repeated over and over again. However, attention-setting takes place in time. Larger changes are likely to take more time, and sets can develop or change over time in an experiment. We are about to enter a new and important dimension.

## 3. Attention-Setting in Time

Time is critical in human thought and behavior, and for attention. Sequential dependencies occur in time, as in the “attentional blink”. This effect comes from an elegant paradigm for studying temporal attention. The method involves a stream of simple stimuli presented one after the other in a single location (rapid serial visual presentation). The method obviates eye movements and allows the researchers to focus on effects of time. In a typical version, the stimuli are single characters that appear for a 10th of a second (100 ms) in the same location; most stimuli are single digits, but a letter appears twice in the stream, and observers are to remember each letter. Observers get set to “grab” the letters from the stream and put their name in working memory. The first letter target is fairly easy to grab, and performance for it is high (typically above 85%). However, while that target is encoded and stored, there is a huge “blink”, during which a second letter is missed as much as 60% more than the first letter (e.g., [84,85]). There is now a large, rich literature on the attentional blink [86]. The deficit for a second target is largest at about 200 to 300 ms after the target and gradually recovers up to 500 ms. Interestingly, a second target can sneak into encoding and memory soon after the first target; it appears to enter with the first target’s set. This “sparing” of temporally close second targets gets stronger when observers can adopt a “grab-several set”, creating “room” in memory (resources) for 2 or 3 items [87]. 

The attentional blink is a sequential dependency that plays out in time; the second target suffers only because a first target preceded it and was attended to. In their thorough review of the blink literature, Dux and Morais [86] argue that no single mechanism can explain the collection of experimental results. The effects seem to involve multiple processes and limitations. At the start of a trial, participants use a target’s visual and semantic features to form an attentional template, which is used for selecting and enhancing targets when they occur. The attentional template can be fairly high-level, specifying object- and even scene targets (e.g., [88]). Subsequent processes encode a selected target, including its name code and a context in working memory; this helps resist replacement by the next stimulus. There is also active evaluation and inhibition of distractors, and response processes [86]. This research area remains active, with elegant recent work on neural and mathematical bases [89,90]. 

In the blink paradigm, the participant must rush to deal with simple but fleeting items. If we expand the time scale to seconds, then larger and more meaningful sets can be instantiated and deeper and more profound sequential dependencies arise. We first illustrate the issues and then turn to the evidence.

### 3.1. Information and Attention over Seconds

The seconds time scale was noted as important because human behavior often plays out over seconds [1]. Moreover, ongoing interactions between the perceiver and the world become apparent [1]. Attention-setting can be central in these interactions because the settings determine the information that humans pick up from the environment. This can produce a profound effect on subsequent behavior because picked-up information can become understanding and learning but *only* if picked up [1]. If information is missed because attention was set differently, there is no understanding or learning.

Consider attention-setting during everyday perception in a public square. Although observers do not usually shout this, “there are so many tasks to do!” Tasks include people to watch and identify, sculptures and fountains to appreciate, and a multitude of events at various time scales to perceive and monitor. The observer can set the task set to “open” and see many things, or adopt an infinite number of more restrictive but sensitive sets. Appreciating live theater in the square requires a continuing, high-level set for perception and comprehension of that event. In contrast, watching birds steal food requires a finer temporal set (those birds can be quick). Additionally, practiced bird watchers, who know what to “look for” in bird behavior, will likely detect the crime before amateurs because knowledge helps guide (set) attention. However, watching birds attentively will reduce attention to the play.

Neisser argued that there was a continuing interaction between the perceiver’s knowledge and attention on one hand, and the information in the world on the other. Perceivers bring differing knowledge to a situation and set their attention differently. A theater lover might arrange their picnic with a great view of the play but not notice the birds until it is too late. A bird watcher will be able to anticipate and follow the theft of food. An individual who loves theater *and* knows about birds will search for protective cover (overhead wires work) before opening the picnic basket and enjoying the show.

Attentional sets cause temporal dependencies; the set at one moment determines what information is picked up from the world, and the extracted information can then be processed to become meaning for the perceiver. The new meaning can then guide further pick-up of information. In theater, early acts set up themes to attend to in later acts. This is a sequential dependency; the later acts will be understood fully only if the early information had been attended to. The contingent pick-up of information is a sense in which perception and understanding are constructed by attention.

### 3.2. Evidence from the Seconds Time Scale

The missed gorillas and shapes show that when participants are set for one task, unexpected information can be missed [29,58,63]. The missed gorilla walked and thumped its chest for over 5 s in the middle of the video [58]. Most et al. [29] treated the missing of unexpected stimuli in terms of information pick up and Neisser’s [1] theory, and this led to the prized large effects discussed earlier. In this research, attention settings determine what is picked up over seconds. However, there has been relatively little follow-up research at the seconds timescale. A major challenge is that there are multiple mental processes taking place over seconds, reducing experimental control. Researchers in attention tend to favor experiments that isolate particular processes, and that is more easily done at sub-second timescales (<300 milliseconds).

Research in other areas of cognition demonstrates the importance of how attention is set. There is considerable research on interactions over seconds in language comprehension. For example, in understanding the meaning of passages, it greatly helps to have an appropriate title to set up comprehension processes. If comprehension processes are not set up properly, perception, comprehension, and learning are slowed and can fail [91,92]. More generally, scaffolding can set up more effective attention, learning, and problem solving [93]. Understanding a play may involve an event-model that, once set up, guides attention and eye fixations [94]. Diagrams for problem representation can be designed to guide attention more effectively over seconds, increasing the rate of problem solving [95]. Setting attention influences what problem-solvers attend to, which can improve their solutions [96]. Similarly, weather map displays can be designed to optimize bottom-up information in ways that interact positively with top-down knowledge, facilitating inference processes that take place over seconds [97].

Attention over time is critical for understanding real-world behaviors such as driving. There is now a sizeable literature on attention and driving, and the interactions of tasks, distraction, and driving hazards (e.g., [98,99]. In-vehicle distractions such as using a navigation system compromise attention to the road for many seconds [99]. In fact, Strayer et al. [99] found that deficits continue even after the distracting task is completed, for up to 27 s.

In more basic attention research, the interplay of set and perception has been examined in the large body of research on task switching, but usually at sub-second time scales. When participants have completed one simple task, it is relatively easy to do that task again with a new stimulus. If the task changes, it takes a fair amount of time (several 10ths of a second) to set up another familiar task and perform it [59,60,61]. As noted, task-switching processes are complex. This is true even when the stimuli and tasks are generally simple (e.g., switches between a parity task, *is 8 odd or even?* and a magnitude task, *is 8 more or less than 5*). A number of interesting experimental designs have been developed, and numerous processing systems have been implicated in this research, as well as large practice effects and a good amount of flexibility [59,60,61]. However, conflict is often created by design because it increases the effect size to a greater level. In the digit-tasks example, a digit activates two conflicting task-interpretations and responses, complicating processing and task switching. Appreciating the full power of task switching in the brain may require complex tasks that do not confuse the brain about what to do.

Research on task switching with complex displays that change over seconds is beginning (e.g., [23,100]). Sanocki and Sulman [23] designed a dynamic task-switching situation from the ground up, to examine perceptual efficiency as task sets are instantiated and changed. This research produced large effects over seconds, and inklings of profound effects. We now describe the research in some detail.

**Figure 1:**
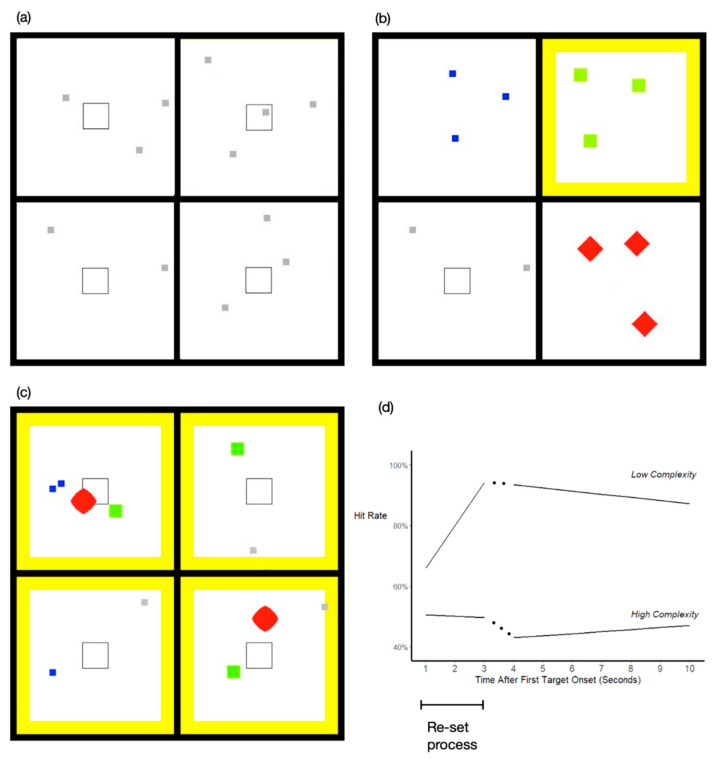
(**a**–**c**) Snapshots from 3 conditions: (**a**) single-task, (**b**) multiple-task grouped, and (**c**) mixed multi-task. The timing and location of the tokens appeared random, but were structured by algorithm; thus the tokens in the figures are at different stages in their lifetimes. (**d**) shows lines fit to data from a single-task condition (“low complexity” on top, from Experiment 3), and the highest complexity condition (“high complexity” on bottom, from Experiment 2, mixed multi-tasking). Data were fit separately for Times 1–3 s and 4–10 s.

To produce complex dynamic displays, Sanocki and Sulman used changing objects as the elements and presented many of them during trials that lasted 60 s. Figure 1a–c show three time slices, from different task conditions. In every condition, all four quadrants were relevant. The small objects were the individual elements (object tokens); each one “lived” over a period of 4 s: the token appeared (“onset”), changed, and then offset over that period. A total of 144 tokens appeared during the trial. The observers’ task was to monitor these tokens and look for targets, which changed more than distractors. For example, in the color task, each token onset was green-yellow, then became more yellow, and then changed back to green-yellow. Distractors changed some toward yellow but a target changed more so (to match the yellow border in the figure). Observers tried to share attention among all of the active tokens but shift attention to detect a token changing strongly (i.e., a target). Since all four quadrants were relevant, observers had to continually shift their attention and eyes. Depending on the condition, there was a total of 4 different tasks that could occur, each with its own distinct object-type. The most basic contrast in the experiments was between single-tasking conditions (Figure 1a; one task throughout the display), and multi-tasking conditions, with a different task in each quadrant (Figure 1b). Training occurred at the beginning of the session. Further details and a description of typical trials are provided below.

Details and Illustration of Method
Before the test period, participants were trained with the four tasks and their distinct object-types. Only one token was shown at a time during training. Observers learned each task to near perfection. Then testing began, with many tokens on each trial. The tasks are described next, but first we describe a typical trial. At the start of a trial, the four (empty) quadrants were shown until the observer pressed a spacebar. Then, object tokens started to appear, one at a time but several per second, until reaching the maximum of 12 active tokens. Each token was offset at the end of its lifetime of 4 sec, and a replacement token would soon appear. During the 60 sec trials, there was a total of 136 distractor tokens and 8 target tokens, distributed throughout the quadrants. Observers were instructed to respond only to targets. On single-task trials, observers would see only one object-type and one task in all four quadrants (e.g., Figure 1a). There were two multi-task conditions used in the experiment. During the first type, there was one type of a different task in each quadrant (4 tasks in total; Figure 1b) but otherwise the same timing parameters as single-task trials. The second type (Figure 1c) will be explained below.
The four tasks were thought to require different processes in the brain, and they were distinguished by their distinct object tokens. The color task had square tokens changing from green-yellow to yellow and back, as mentioned. The shape task had red tokens changing in concavity, from fat-diamonds to concave (star-like) and back. The location task had grey squares moving linearly (and bouncing off walls); targets passed through the central square outline (“more” is defined as the proximity to the central square). Finally, in the motion task, the blue squares moved left to right with up/down deviations; the targets moved up or down more, as if drunk.

We began with the basic single versus multiple task question: are participants more efficient when they continually perform one task, compared to continually switching between four tasks? Performance was measured well by the hit-rate for detecting targets, and the average performance for single-task conditions was compared to the four-task condition. Since the four-task condition required continual task switching, we expected a large advantage for the single-task conditions. The average single-task hit rate was 78.4%, and the multi-task hit rate was 14.1% lower. This difference (multi-task cost) was highly reliable and moderately large. However, it was not yet the catastrophic deficit expected when complexity became too high. The result suggests that in the multi-task condition, participants are learning to switch between tasks with a fair degree of efficiency, although less than in single-task conditions.

The potentially profound results involve the preparation of efficient processing over time. Preparation effects were found previously (Sulman and Sanocki, unpublished) and were replicated and expanded here. After participants detected one target during the trial, there was a deficit for other targets appearing during the next few seconds. Like the attentional blink, this effect was a sequential dependency. In the present case, detecting and responding to one target likely requires time and effort, as does the resumption of processes that monitor tokens. The detection of new targets suffers as a result. The lower task-complexity conditions produced the largest preparation effect because efficient processing was re-set over 3 s. This is illustrated by the top function in Figure 1d (low complexity). The hit rates were fit to lines, separately for the first 3 data points, and then for seconds 4 through 10. As can be seen, the hit rate was relatively low 1 sec after the first target and increased until 3 s because efficient processing was re-set. (Actual data points and their variability are shown in [23].) This result suggests that there is a set-up process that must be completed for optimal efficiency to be achieved. The low-complexity function shown is for single-tasking. However, even in the first multi-tasking condition (Figure 1b), observers were also able to re-set for efficiency over approximately 3 sec, if the response was not complex. (Observers re-set at rates similar to single-tasking but reached a lower asymptote of efficiency; see [23].)

Sanocki and Sulman [23] further increased the complexity, in search of a catastrophic breakdown. Combining two complexity manipulations in a second type of multi-tasking condition resulted in a breakdown, and a complete elimination of efficient set-up. This result is shown in the bottom function in Figure 1d (highest complexity). Processing efficiency never increased after a target detection; the observers lost their ability to become efficient. In contrast, the same observers were able to set up efficiency in the less complex single-tasking condition. The elimination of effective re-setting in the highest complexity condition is a potentially profound result.

The two complexity manipulations and the results will now be explained. The first manipulation involved the stimuli; instead of grouping objects (tasks) by quadrant, the objects (tasks) were mixed together in the display space (Figure 1c). This raised the costs of multi-tasking considerably; the overall deficit compared to single-tasking was now 34.1% compared to 14.1%. Furthermore, the re-setting of the efficient performance was slowed (data in [23]). The overall results show that multiple tasks are handled more efficiently when the four tasks are spatially segregated (as in Figure 1b) than when not (Figure 1c). Spatial organization may be a principle of human behavior; we group tasks in the home, for example, putting cooking in one room, relaxing in another, and private behaviors in still others.

The second complexity manipulation was the response rule, which could be simple or complex in different experiments. (In the simple-response experiments (lower complexity), participants pressed a button whenever a target occurred. In the complex-response experiments (higher complexity), participants had to indicate the quadrant that a target appeared in by pressing one of four corresponding buttons.) As response complexity increases, additional attentional resources are presumed to be necessary for encoding and responding to a target, leaving fewer resources for re-setting attention. The re-set process occurred more slowly in the complex response conditions than in the simple conditions.

When the difficult mixed condition was combined with complex responses, performance reached its lowest level (the 34.1% deficit mentioned relative to the single-tasking control). Additionally, and perhaps most significantly, when the time course of re-setting was examined (“highest complexity” in Figure 1d), there was no re-setting at all in this difficult condition and no rise over time after a target detection. One could say that the conditions were so challenging that attention could not properly set up efficient processing. These large deficits were found throughout the session, over many trials, and constituted repeated missed gorillas. Yet, when the same observers were in single-task conditions, their attention was able to set up efficient processing over seconds.

The results are consistent with the claim that attention-setting is an essential top-down process that takes place over time. Human goals such as detecting targets efficiently in a complex world require the preparation and set up of mental processes over seconds. The results are relevant to real-world functioning because human behavior often plays out over seconds. Complex tasks can take seconds or more to set up. When tasks become too difficult, there may be a complete breakdown in comprehension, with major negative consequences. For example, learners in school may become overwhelmed when the material is too difficult, or soldiers in battle may become overwhelmed by multiple critical threats; in each case, the cognitive overload can result in a complete task failure such as that in the high-complexity condition. Further behavioral research at the seconds timescale should be illuminating. Recent work in our lab with dynamic displays and the seconds timescale has found further large effects on task set-up with known tasks. The results are another example of contingent pick-up. When observers were set for one task, they missed information about a task change for many trials, reducing performance with the new task markedly (Sanocki and Lee, in preparation).

## 4. The Biological Attention-Setting Machine

We now round out the big picture with some relevant findings from neuroscience and cognitive health. Neuroscientists have developed incisive methods and are beginning to apply them in situations that approach real-world complexity at the seconds timescale. In particular, Crittenden, Mitchell, and Duncan led a program of research that captured attention-setting machinery in the brain with complex tasks. They used fMRI methods, which measure the time scale of seconds, because the integration of the signals over seconds is necessary for reliability. In [101], participants switched between six different tasks, and a large task difference was (for example) a switch between a knowledge-task and a spelling task (*Is this object (picture) a living thing* versus *Does A fit H_VE to make a word?*). The researchers found that major brain networks were active during these large switches but much less so with small task-switches, between more similar tasks.

The research team has identified a major brain network that underlies task switching, which they term the multiple demand network (e.g., [101,102,103]). The multiple demand (MD) network serves to connect and guide processing while completing the tasks. It binds the task set, including task-relevant cognitive fragments such as memory registers, integrations of relevant stimulus inputs, task rules, appropriate knowledge, and potential responses and actions, while inhibiting irrelevant processing. The network is coordinated and general-purpose, hence, the name *Multiple Demand*. The parts are active in a variety of different tasks, and their activity levels are correlated across the tasks. In other words, these regions appear to be programmable for different tasks, serving as multiple-use computational space. The network includes more specialized regions; however, the specialized regions are most apparent only when the tasks are easy and the attentional load is low [103]. When the task demands are high, due to task complexity or time pressure, the general portions of the MD network become more active and more tightly interconnected. This allows for rapid communication within the entire network [103]. Moreover, under pressure the general MD network expands in neural extent, increasing in size by spreading more into the frontal brain (anterior spread; [103]).

In summary, major portions of the MD network are dynamically allocated general computational space. When a task is complex, such as watching a theatrical play, attention may work by setting up processing in the MD space, including connections to more specialized regions for language and perhaps drama. The MD network can expand into rental space (into added areas of cortex) in case of bird attacks. The MD network may be the main implementation level of attention-setting, in the Marrian sense [104]. The set-up processes often takes place over seconds. The better perception (and the comprehension and learning) that can result is a way in which perception is actively constructed.

One could say that attention-setting and executive processing are organic functions somewhat like a muscle; a muscle’s strength is built up through active use, and it can get weak through fatigue or dis-use. Aerobic exercise increases blood flow to the brain and strengthens executive processing while protecting against the negative effects of aging (e.g., [105,106,107]). However, while using attention is good in general, continual overuse due to chronic stress may not be healthy (e.g., [108]). One might imagine the MD network starting to let off steam or burning oil.

Even periods of healthy mental exercise, such as normal hard work, can result in temporary mental fatigue. Although not damaging, fatigue does reduce the ability to set attention subsequently [109]. Fortunately, research is also beginning to document ways to restore attention. These include the relaxed, pleasant use of attention (“gentle fascination”), removed from strong demands [109,110], as well as methods of meditation (e.g., [111]. This research is part of a larger goal of developing guidelines that encourage human flourishing, including the healthy functioning of the brain, the self, and attention [112].

## 5. Conclusions

Attention research brings together multiple perspectives and disciplines. Here, we proposed a mental action framework for understanding top-down attention: attention-setting is a process of setting up and prioritizing brain functions in the service of intentions and goals. Attention-setting causes large effects in human performance, as reviewed. Appropriate attention-setting can result in highly efficient selective perception and learning, whereas inappropriate settings can prevent it. This is a major way in which the brain is “active” and perception is constructed. The attention-setting framework can explain major attention phenomena. However, much further specification of the framework is needed, including research on the potentially profound effects over time.

We argue that attention-setting is not a clearly defined “part” of the brain; its workings are functionally integrated with other mental processes, including basic perception and memory. Attention-setting is most critical in complex human situations, and recent neuroscience research is beginning to chart this functional network in the brain [102,103]. The high complexity of attention-setting in real-world situations invites and even requires the use of powerful research tools, including computational programs involving sets of networks (e.g., [6]). Now is a great time for research on the complexities of attention, and an exciting time for integrative brain research in general.

## Data Availability

The data presented here are available from the authors.
